# Abemaciclib plus fulvestrant in hormone receptor-positive, human epidermal growth factor receptor 2-negative advanced breast cancer in premenopausal women: subgroup analysis from the MONARCH 2 trial

**DOI:** 10.1186/s13058-021-01463-2

**Published:** 2021-08-23

**Authors:** Patrick Neven, Hope S. Rugo, Sara M. Tolaney, Hiroji Iwata, Masakazu Toi, Matthew P. Goetz, Peter A. Kaufman, Yi Lu, Nadine Haddad, Karla C. Hurt, George W. Sledge

**Affiliations:** 1 Department of Oncology, University Hospitals Leuven, KU Leuven-University of Leuven, Herestraat 49, 3000 Leuven, Belgium; 2 Department of Medicine (Hematology/Oncology), University of California San Francisco Helen Diller Family Comprehensive Cancer Center, San Francisco, CA USA; 3 Department of Medical Oncology, Dana-Farber Cancer Institute, Boston, MA USA; 4 Department of Breast Oncology, Aichi Cancer Center Hospital, Nagoya, Japan; 5 Department of Breast Surgery, Graduate School of Medicine, Kyoto University, Kyoto, Japan; 6 Department of Oncology, Mayo Clinic, Rochester, MN USA; 7 Department of Hematology and Oncology, Norris Cotton Cancer Center at Dartmouth-Hitchcock Medical Center, Lebanon, NH USA; 8 Eli Lilly and Company, Indianapolis, IN USA; 9 Department of Medicine, Stanford University School of Medicine, Stanford, CA USA

**Keywords:** Abemaciclib, Advanced breast cancer, CDK4 and 6 inhibitor, Fulvestrant, Premenopausal women

## Abstract

**Background:**

In MONARCH 2, abemaciclib plus fulvestrant significantly improved median progression-free survival (PFS, 16.4 vs 9.3 months, hazard ratio [HR] 0.553) and overall survival (OS, 46.7 vs 37.3 months; HR 0.757) compared with placebo plus fulvestrant in hormone receptor-positive (HR-positive), human epidermal growth factor receptor 2-negative (HER2-negative) advanced breast cancer (ABC) patients who were endocrine therapy (ET) resistant, regardless of menopausal status. Here, we report findings in the premenopausal subgroup of the MONARCH 2 trial.

**Methods:**

The premenopausal subgroup included patients with natural menstrual bleeding who received a gonadotropin-releasing hormone agonist at least 4 weeks prior to study treatment start date and for the entire study duration. Of the 669 patients enrolled in the MONARCH 2 trial, 114 were premenopausal (abemaciclib plus fulvestrant, *n* = 72; placebo plus fulvestrant, *n* = 42), and were included in this analysis. The primary objective was investigator-assessed PFS and secondary objectives were OS, objective response rate, and safety and tolerability. Exploratory analyses included time to second disease progression (PFS2), time to chemotherapy (TTC), and chemotherapy-free survival (CFS).

**Results:**

At the primary objective cutoff (February 14, 2017), median PFS was not reached for the abemaciclib plus fulvestrant arm versus 10.52 months for the placebo plus fulvestrant arm (HR 0.415; 95% CI 0.246–0.698). At the pre-specified OS interim cutoff (20-June-2019), median PFS was 28.6 months in the abemaciclib plus fulvestrant arm compared with 10.26 months in the placebo plus fulvestrant arm (HR 0.477; 95% CI 0.302–0.755). A numerical OS benefit was observed with abemaciclib plus fulvestrant compared to fulvestrant alone (HR 0.689; 95% CI 0.379–1.252, median, not reached vs 47.3 months). Improvements were also observed for the exploratory outcomes of PFS2 (HR 0.599), TTC (HR 0.674), and CFS (HR 0.642) with the addition of abemaciclib to fulvestrant. The safety profile was generally consistent with results disclosed previously.

**Conclusions:**

Results of the premenopausal subgroup in the MONARCH 2 trial were consistent with the improved clinical outcomes observed in the intent-to-treat population. The analysis provides support for the use of abemaciclib plus fulvestrant (with ovarian suppression) as an effective treatment option for premenopausal patients with HR+, HER2- ABC who are ET-resistant.

*Clinical trial registration*: NCT02107703. Registered April 08, 2014- Retrospectively registered, https://clinicaltrials.gov/ct2/show/NCT02107703.

**Supplementary Information:**

The online version contains supplementary material available at 10.1186/s13058-021-01463-2.

## Background

In recent years, the incidence of advanced breast cancer (ABC) in women aged 25 to 39 years has slightly increased [1] and the disease is often more aggressive, with a poorer prognosis, and high rates of recurrence and mortality [2, 3]. Hence, there is growing interest in appropriate treatment strategies for pre-/perimenopausal women (hereafter referred to as premenopausal women) with hormone receptor-positive (HR +), human epidermal growth factor receptor 2-negative (HER2-) ABC [2, 4–6]. This population subgroup is often under-represented in clinical trials [3, 6, 7].

An optimal treatment approach in young women with HR+, HER2- ABC is still poorly defined and treatment strategies are usually derived from data from postmenopausal patients. Studies have highlighted that given the differences in tumor biology and quality of life, extending treatment options across patients with different menopausal status might not be the best strategy [2, 7, 8]. Additionally, there is the potential that these young women are at risk of over-treatment, based solely on age considerations [9].

Cyclin-dependent kinase 4 and 6 (CDK4 and 6) inhibitors have led to substantial improvements in progression-free survival (PFS) in the ABC setting, resulting in approval of CDK4 and 6 inhibitors in combination with ET or as monotherapy in ABC patients [10–14]. More recently, some studies have also demonstrated overall survival (OS) benefit, further establishing the effectiveness of these agents in the treatment of ABC [6, 15, 16]. Some of these [4, 5, 15] reported improved PFS in this premenopausal subgroup, leading to the recommendation of fulvestrant in combination with CDK4 and 6 inhibitors and ovarian suppression as a treatment option for premenopausal patients with HR+, HER2- ABC [17].

Abemaciclib is an oral, selective, and potent small molecule CDK4 and 6 dual inhibitor for the treatment of HR+, HER2- ABC that is dosed on a continuous schedule. Abemaciclib is currently approved in three indications in HR+, HER2- ABC; in combination with aromatase inhibitor for postmenopausal women as initial endocrine-based therapy (MONARCH 3) [12, 14], in combination with fulvestrant for women with disease progression following ET (MONARCH 2) [13], and as a single agent for patients with disease progression following ET and prior chemotherapy in the metastatic setting (MONARCH 1) [12, 14].

In the intent-to-treat (ITT) population (669 patients) of the MONARCH 2 trial, abemaciclib plus fulvestrant significantly improved investigator-assessed PFS compared with placebo plus fulvestrant in women with HR+, HER2- ABC who were ET-resistant (median PFS, 16.4 vs 9.3 months; hazard ratio [HR], 0.553; 95% confidence interval [CI], 0.449–0.681*; P* < 0.001) [13]. Abemaciclib treatment resulted in improved objective response rate (ORR; 48.1% vs 21.3% in the control arm). The OS was significantly improved in the abemaciclib plus fulvestrant arm (median OS, 46.7 vs 37.3 months; HR 0.757; 95% CI 0.606–0.945; *P* = 0.01) [15]. The addition of abemaciclib to ET led to consistent treatment benefit across subgroups including menopausal status. Here, we report the efficacy and safety results for the premenopausal patient subgroup from the MONARCH 2 trial.

## Methods

### Study design and patients

MONARCH 2 was a randomized, double-blind, phase III, trial of abemaciclib or placebo with fulvestrant in HR+, HER2- ABC women with any menopausal status (pre-, peri-, or post-menopausal) who had disease progression following ET. Patients were required to have disease that progressed while receiving prior ET (neoadjuvant or adjuvant ET, ≤ 12 months after completion of adjuvant ET, or while receiving first-line ET for metastatic disease). Patients with more than one ET or any prior chemotherapy for ABC were excluded. Full trial details and eligibility criteria were published previously [13].

This article presents the subanalysis of the premenopausal population which comprised patients with natural menstrual bleeding who received a gonadotropin releasing-hormone (GnRH) agonist, such as goserelin, at least 4 weeks prior to study treatment start date and for the entire study duration.

The study was approved by the ethical and local institutional review boards for the sites participating in the clinical trial, and was conducted in accordance with the Good Clinical Practice guidelines and the Declaration of Helsinki. All patients provided written informed consent before enrollment. This study was overseen by a steering committee, and safety data were evaluated quarterly by an independent data monitoring committee.

### Treatment procedure

Details of the MONARCH 2 trial have been published previously [13]. Briefly, patients received abemaciclib (150 mg) or placebo twice daily during each 28-day cycle plus 500 mg fulvestrant (per label). Treatment continued until progressive disease, death, or discontinuation for any other reason.

### Efficacy and safety assessments

Safety analysis was performed on all patients who received at least one dose of study drug. Adverse events (AEs) were graded according to the National Cancer Institute Common Terminology Criteria for Adverse Events (CTCAE), version 4.0, and were evaluated at every patient visit from baseline until follow-up.

### Endpoints

The primary endpoint was investigator-assessed PFS as defined by Response Evaluation Criteria in Solid Tumors version 1.1, and measured from the time of randomized assignment until progressive disease or death (whichever was earlier). The secondary endpoints included OS (time of randomized assignment until death), ORR (proportion of patients with complete response or partial response [PR]), and safety and tolerability. Exploratory endpoints included: PFS2, defined as time from randomization to discontinuation of first subsequent post-discontinuation therapy or death (whichever was earlier), TTC, defined as time from randomization to initiation of first post-discontinuation chemotherapy (censoring patients who died prior to initiation of chemotherapy), and CFS, defined as time from randomization to initiation of first post-discontinuation chemotherapy or death (whichever was earlier).

### Statistical analyses

The data cutoff for the primary analysis of PFS occurred on February 14, 2017. ORR, change in tumor size, and safety evaluations were also reported from the primary analysis. The data cutoff for the preplanned interim analysis of the key secondary endpoint of OS occurred on 20 June, 2019. At this interim data cutoff, PFS was updated and the exploratory endpoints of PFS2, TTC, and CFS were also evaluated descriptively.

All efficacy analyses were performed on the subgroup of premenopausal patients within the ITT population, corresponding to all premenopausal patients randomized to study treatment. For time-to-event endpoints, the Kaplan–Meier method was used to estimate the survival curve for each treatment group, and the Cox proportional hazard model was used to estimate the HR and corresponding 95% CI with an interaction term of menopausal status with treatment group. An exploratory mixed-model analysis was used to compare change in tumor size over time. Unless otherwise noted, all confidence intervals used a 95% confidence level. Safety was assessed in all premenopausal patients who received at least one dose of any study treatment (i.e. the safety population). The statistical analyses were performed using SAS (version 9.2 or later; SAS Institute, Cary, NC).

## Results

### Patients and treatment

In the MONARCH 2 trial, 669 patients were randomly assigned to receive abemaciclib plus fulvestrant (*n* = 446) or placebo plus fulvestrant (*n* = 223). Of these, 114 patients (17.0%) were premenopausal (premenopausal population) and received ovarian suppression with a GnRH agonist. This included 72 patients in the abemaciclib plus fulvestrant arm, of whom 71 received study treatment, and 42 in the placebo plus fulvestrant arm.

Demographic and baseline characteristics were well-balanced between the two treatment arms (Table 1). At baseline, 60 patients (52.6%) presented with visceral disease and 34 (29.8%) with bone-only disease. A total of 43 patients (37.7%) had primary ET resistance (defined as relapse while on the first 2 years of adjuvant ET or PD within the first 6 months of first-line ET) [18].

**Table 1:** Patient and disease baseline characteristics for premenopausal patients in the MONARCH 2 trial

	Abemaciclib + fulvestrant *N* = 72	Placebo + fulvestrant *N* = 42
Median age (range)	46 (32–57)	47 (32–66)
Race, *n* (%)		
Asian	51 (70.8)	24 (57.1)
Caucasian	14 (19.4)	16 (38.1)
Other	7 (9.7)	2 (4.8)
Most recent ET, *n* (%)^a^		
Neoadjuvant or adjuvant	44 (61.1)	21 (50.0)
Metastatic	26 (36.1)	20 (47.6)
Number of lines of ET, *n* (%)		
1	60 (83.3)	30 (71.4)
2	10 (13.9)	11 (26.2)
Prior AI, *n* (%)		
Yes	10 (13.9)	12 (28.6)
No	62 (86.1)	30 (71.4)
Sensitivity to ET, *n* (%)^a^		
Primary resistance^b^	28 (38.9)	15 (35.7)
Secondary resistance^c^	42 (58.3)	26 (61.9)
Progesterone-receptor status, *n* (%)		
Positive	54 (75.0)	38 (90.5)
Negative	18 (25.0)	4 (9.5)
Metastatic site, *n* (%)		
Visceral	43 (59.7)	17 (40.5)
Bone only	19 (26.4)	15 (35.7)
Other	10 (13.9)	10 (23.8)
Measurable disease, *n* (%)		
Yes	51 (70.8)	28 (66.7)
No	21 (29.2)	14 (33.3)
ECOG performance status		
0	54 (75.0)	36 (85.7)
1	18 (25.0)	6 (14.3)

AI, aromatase inhibitor; ECOG, Eastern Cooperative Oncology Group; ET, endocrine therapy

^a^Two patients in the abemaciclib arm and one patient in the placebo arm received no prior ET; ^b^Patients whose disease relapsed ≤ 2 years while receiving (neo) adjuvant ET or progressed ≤ 6 months of receiving ET for ABC; ^c^Patients receiving prior ET who do not meet the definition of primary resistance were considered to have secondary resistance

At the primary PFS cutoff, a total of 40 (55.6%) patients in the abemaciclib plus fulvestrant arm and 11 (26.2%) patients in the placebo plus fulvestrant arm remained on treatment. The median number of cycles received was 17 in the abemaciclib plus fulvestrant arm and 11 in the placebo plus fulvestrant arm. At the data cutoff for interim OS analysis, 21 premenopausal patients (29.2%) in the abemaciclib plus fulvestrant arm versus four patients (9.5%) in the placebo plus fulvestrant arm were still receiving the study treatment. The median length of follow-up was approximately 4 years in each treatment arm (48.69 and 47.70 months in the abemaciclib plus fulvestrant and placebo plus fulvestrant arms, respectively).

### Progression-free survival

**Fig. 1:**
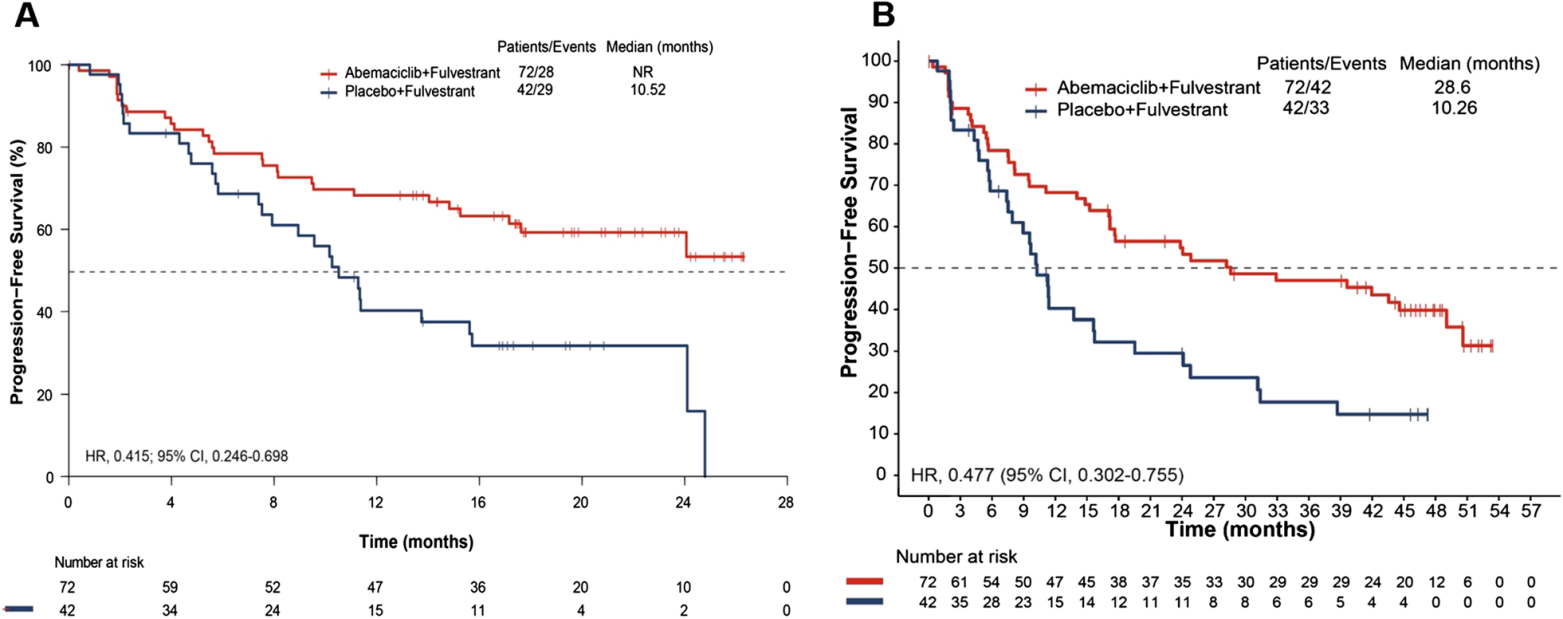
Kaplan–Meier curves for progression-free survival in the premenopausal population of the MONARCH 2 trial **A** PFS at primary analysis, **B** Updated PFS at interim analysis. CI, confidence interval; HR, hazard ratio; NR, not reached; PFS, progression-free survival

At the time of the primary analysis cutoff, 28 (38.9%) versus 29 (69.0%) PFS events were observed and mPFS was not reached versus 10.52 months in the abemaciclib plus fulvestrant and placebo plus fulvestrant arms, respectively. The addition of abemaciclib to fulvestrant resulted in a meaningful improvement in PFS (HR 0.415; 95% CI 0.246–0.698), Fig. 1A. The PFS benefit was confirmed by blinded independent central review (HR 0.432; 95% CI 0.236–0.793).

At the interim OS analysis cutoff, 20 June 2019, the PFS analysis was updated. In the abemaciclib plus fulvestrant arm, 42 (58.3%) PFS events were observed versus 33 (78.6%) in the placebo plus fulvestrant arm. The median PFS achieved in the abemaciclib plus fulvestrant arm was 28.6 months compared with 10.26 months in the control arm (HR 0.477; 95% CI 0.302 to 0.755), Fig. 1B.

### Objective response rate and tumor shrinkage

ORR was higher in the abemaciclib plus fulvestrant arm compared to the placebo plus fulvestrant arm (43.1%; 95% CI 31.6–54.5 vs 19.0%; 95% CI 7.2–30.9) (Table 2). This included two (2.8%) complete responses in the abemaciclib plus fulvestrant arm compared with no complete response in the placebo plus fulvestrant arm. Patients with measurable disease achieved an ORR of 60.8% (95% CI 47.4–74.2) in the abemaciclib plus fulvestrant arm and 28.6% (95% CI 11.8–45.3) in the placebo plus fulvestrant arm (*P* = 0.006). An exploratory analysis of mean change in tumor size demonstrated that after 12 cycles, tumor size in the abemaciclib plus fulvestrant arm decreased by 64.6% compared to 42.5% in the placebo arm.

**Table 2:** Best overall response in the premenopausal population of the MONARCH 2 trial

Overall population	Abemaciclib + fulvestrant *N* = 72		Placebo + fulvestrant *N* = 42		*P* value
	*n* (%)	95% CI	*n* (%)	95% CI	
CR	2 (2.8)	− 1.0, 6.6	0 (0)	–	
PR	29 (40.3)	28.9, 51.6	8 (19.0)	7.2, 30.9	
SD	30 (41.7)	30.3, 53.1	27 (64.3)	49.8, 78.8	
≥ 6 months	25 (34.7)	23.7, 45.7	21 (50.0)	34.9, 65.1	
Progressive disease	8 (11.1)	3.9, 18.4	6 (14.3)	3.7, 24.9	
Not evaluable	3 (4.2)	− 0.4, 8.8	1 (2.4)	− 2.2, 7.0	
Overall response rate (CR + PR)	31 (43.1)	31.6, 54.5	8 (19.0)	7.2, 30.9	.009
Disease control rate (CR + PR + SD)	61 (84.7)	76.4, 93.0	35 (83.3)	72.1, 94.6	.845
Clinical benefit rate (CR + PR + SD ≥ 6 months)	56 (77.8)	68.2, 87.4	29 (69.0)	55.1, 83.0	.304

Measurable disease population	Abemaciclib + fulvestrant *N* = 51		Placebo + fulvestrant *N* = 28		*P* value
	*n* (%)	95% CI	*n* (%)	95% CI	
CR	2 (3.9)	− 1.4, 9.2	0 (0.0)	–	
PR	29 (56.9)	43.3, 70.5	8 (28.6)	11.8, 45.3	
SD	11 (21.6)	10.3, 32.9	16 (57.1)	38.8, 75.5	
≥ 6 months	7 (13.7)	4.3, 23.2	12 (42.9)	24.5, 61.2	
Progressive disease	7(13.7)	4.3, 23.2	4 (14.3)	1.3, 27.2	
Not evaluable	2 (3.9)	− 1.4, 9.2	0 (0.0)	–	
Overall response rate (CR + PR)	31 (60.8)	47.4, 74.2	8 (28.6)	11.8, 45.3	.006
Disease control rate (CR + PR + SD)	42 (82.4)	71.9, 92.8	24 (85.7)	72.8, 98.7	.702
Clinical benefit rate (CR + PR + SD ≥ 6 months)	38 (74.5)	62.5, 86.5	20 (71.4)	54.7, 88.2	.768

CI, confidence interval; CR, complete response; ITT, intent-to-treat; N, number of patients in the arm; n, number of patients in each subgroup; PR, partial response; SD, stable disease

^*^Using RECIST version 1.1

### Overall survival

**Fig. 2:**
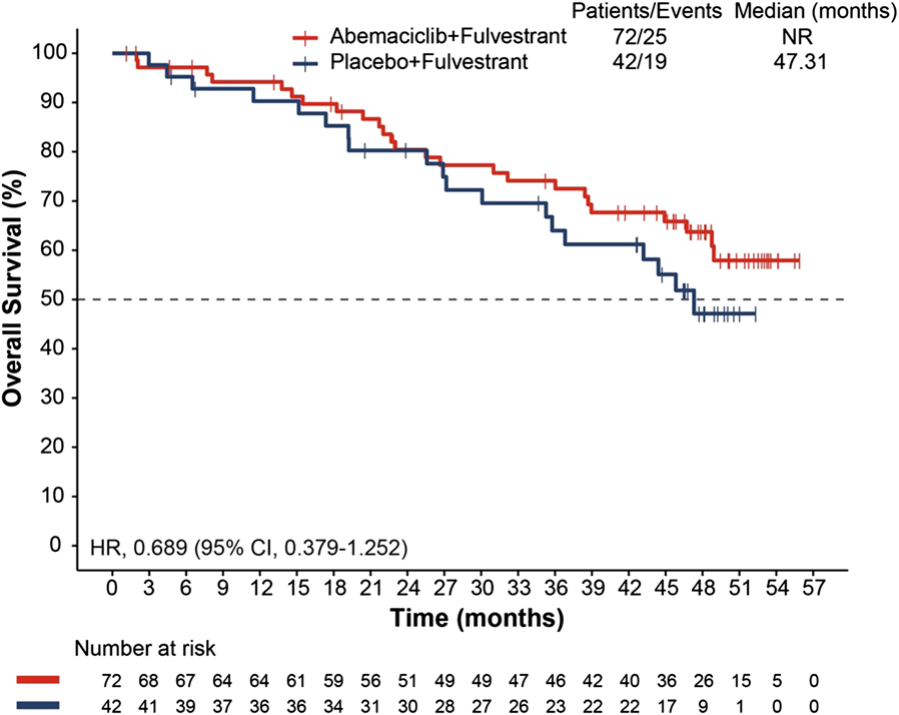
Kaplan–Meier curve for overall survival in the premenopausal population of the MONARCH 2 trial. CI, confidence interval; HR, hazard ratio; NR, not reached

At the interim OS cutoff, there were 25 (34.7%) versus 19 (45.2%) deaths and mOS was not reached versus 47.31 months in the abemaciclib plus fulvestrant versus placebo plus fulvestrant arms, respectively. A numerical OS benefit was observed with the addition of abemaciclib to fulvestrant (HR 0.689; 95% CI 0.379–1.252; Fig. 2) which was consistent with the ITT population.

### Post-discontinuation therapy

At the interim OS analysis, a total of 88 patients had discontinued study treatment including 50 (69.4%) patients in the abemaciclib plus fulvestrant arm and 38 (90.5%) patients in the placebo plus fulvestrant arm. Forty-one (56.9%) patients in the abemaciclib plus fulvestrant arm compared to 35 (83.3%) patients in the placebo plus fulvestrant arm received a post-discontinuation therapy. Chemotherapy was received by 60 patients (*n* = 34, 47.2% patients vs *n* = 26, 61.9% patients in the abemaciclib plus fulvestrant and placebo plus fulvestrant arms, respectively) at any time post-discontinuation. ET was received by 48 patients (*n* = 26, 36.1% patients in the abemaciclib plus fulvestrant arm vs *n* = 22, 52.4% patients in the placebo plus fulvestrant arm), while 41 patients received a targeted therapy (*n* = 19, 26.4% patients in the abemaciclib plus fulvestrant arm vs *n* = 22, 52.4% patients in the placebo plus fulvestrant arm), and 17 patients received other therapies (*n* = 9, 12.5% patients in the abemaciclib plus fulvestrant arm vs *n* = 8, 19.0% patients in the placebo plus fulvestrant arm). Among those who received targeted therapies, 15 patients received CDK 4 and 6 inhibitor(s) as post-discontinuation therapy (*n* = 5, 6.9% patients in the abemaciclib plus fulvestrant arm vs *n* = 10, 23.8% patients in the placebo plus fulvestrant arm).

### Other exploratory endpoints

**Fig. 3:**
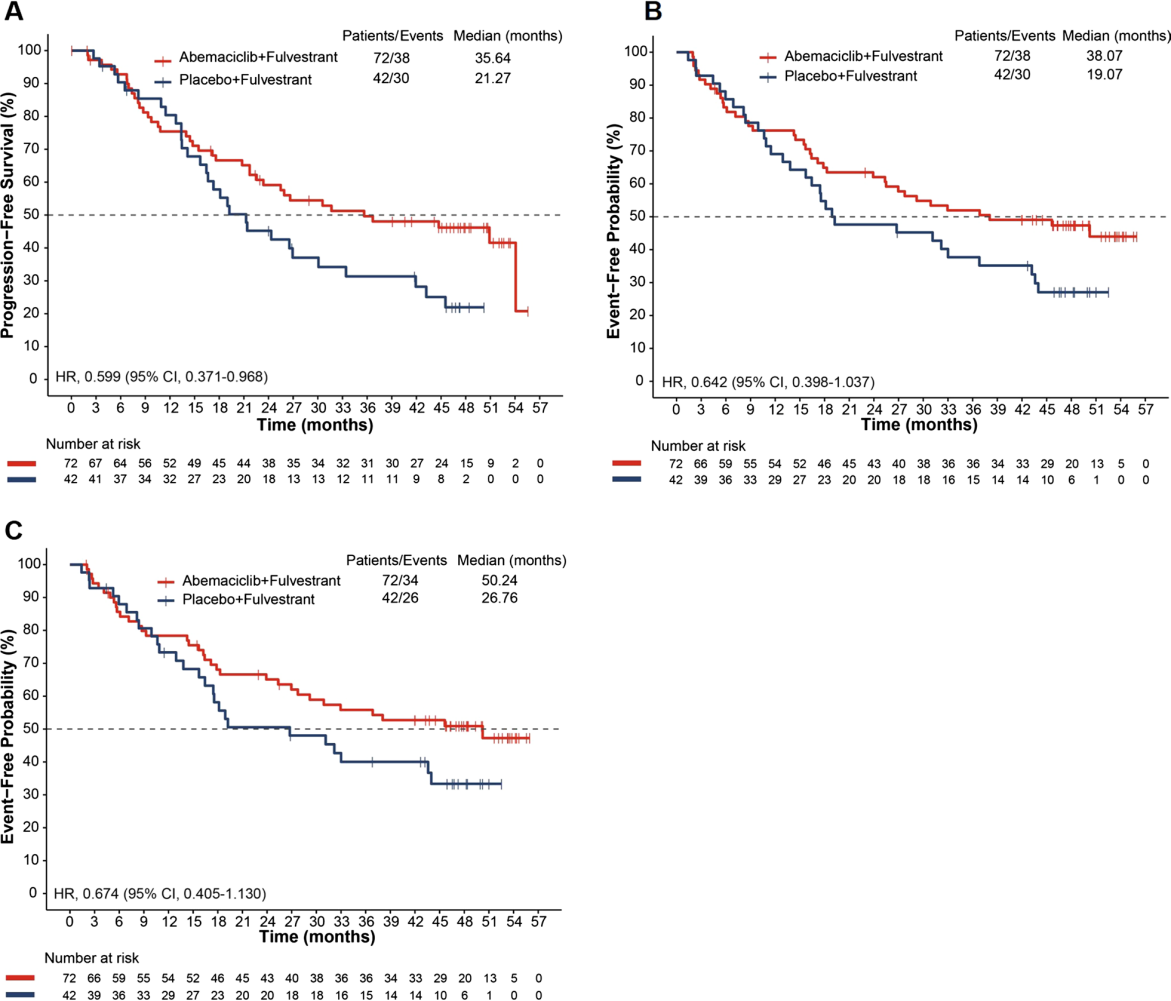
Kaplan–Meier curves for **A** second disease progression, **B** time to chemotherapy, and **C** chemotherapy-free survival in the premenopausal population of the MONARCH 2 trial. CI, confidence interval; HR, hazard ratio

With the addition of abemaciclib to fulvestrant, a clinically meaningful benefit was observed in other exploratory endpoints including PFS2 (HR 0.599; 95% CI 0.371–0.968; Fig. 3A), TTC (HR 0.674; 95% CI 0.404–1.124; Fig. 3B) and CFS (HR 0.642; 95% CI 0.398–1.037; Fig. 3C), consistent with ITT population.

Median PFS2 was 35.64 months in the abemaciclib plus fulvestrant arm versus 21.27 months in the placebo plus fulvestrant arm.

The median time to first chemotherapy treatment (censoring patients who died prior to receiving chemotherapy) was 50.24 months in the abemaciclib plus fulvestrant arm and 26.76 months in the placebo plus fulvestrant arm.

Deaths prior to receiving any chemotherapy were four (5.6%) patients in the abemaciclib arm and 4 (9.5%) patients in the placebo arm. Median CFS (including both chemotherapy and death as events) was 38.07 versus 19.07 months in the abemaciclib versus placebo arms, respectively.

### Safety

The safety profile in the premenopausal subgroup was consistent to the ITT population. The most frequent adverse events of any grade were diarrhea (87.3% vs 23.8%), neutropenia (59.2% vs 7.1%) and leukopenia (43.7% vs 4.8%) in the abemaciclib plus fulvestrant versus placebo plus fulvestrant arms, respectively (Table 3). In the abemaciclib arm, key grade 3 adverse events of special interest were neutropenia (39.4%), leukopenia (16.9%), and diarrhea (11.3%). Serious adverse events were reported in eight patients (11.3%) in the abemaciclib plus fulvestrant arm and two patients (4.8%) in the placebo plus fulvestrant arm (Additional file 1: Supplemental Table 1).

**Table 3:** Treatment-emergent adverse events occurring in ≥ 10% of the premenopausal population of the MONARCH 2 trial

Adverse events (≥ 10% patients in either arm)	Abemaciclib + fulvestrant *N* = 71				Placebo + fulvestrant *N* = 42			
	All	G2	G3	G4	All	G2	G3	G4
Any, *n* (%)	70 (98.6)	20 (28.2)	40 (56.3)	4 (5.6)	40 (95.2)	17 (40.5)	7 (16.7)	0
Diarrhea	62 (87.3)	22 (31.0)	8 (11.3)	0	10 (23.8)	1 (2.4)	0	0
Neutropenia^a^	42 (59.2)	9 (12.7)	28 (39.4)	2 (2.8)	3 (7.1)	1 (2.4)	1 (2.4)	0
Leukopenia	31 (43.7)	15 (21.1)	12 (16.9)	0	2 (4.8)	1 (2.4)	0	0
Infections and infestations	31 (43.7)	26 (36.6)	1 (1.4)	0	11 (26.2)	7 (16.7)	2 (4.8)	0
Abdominal pain	25 (35.2)	2 (2.8)	0	0	5 (11.9)	2 (4.8)	0	0
Anemia	24 (33.8)	15 (21.1)	7 (9.9)	0	1 (2.4)	0	0	0
Headache	24 (33.8)	7 (9.9)	–	0	13 (31.0)	2 (4.8)	0	0
Vomiting	23 (32.4)	5 (7.0)	1 (1.4)	0	3 (7.1)	0	1 (2.4)	0
Nausea	20 (28.2)	6 (8.5)	1 (1.4)	0	10 (23.8)	3 (7.1)	1 (2.4)	0
Respiratory disorders	16 (22.5)	4 (5.6)	1 (1.4)	0	9 (21.4)	1 (2.4)	0	0
Pyrexia	13 (18.3)	0	0	0	6 (14.3)	1 (2.4)	0	0
Stomatitis	13 (18.3)	3 (4.2)	0	0	9 (21.4)	2 (4.8)	0	0
Pruritus	13 (18.3)	1 (1.4)	0	0	2 (4.8)	0	0	0
Thrombocytopenia	12 (16.9)	4 (5.6)	0	2 (2.8)	–	–	–	–
Alanine aminotransferase increased	11 (15.5)	4 (5.6)	3 (4.2)	0	2 (4.8)	0	0	0
Rash	11 (15.5)	2 (2.8)	1 (1.4)	0	–	–	–	–
Constipation	10 (14.1)	1 (1.4)	1 (1.4)	0	7 (16.7)	0	0	0
Arthralgia	10 (14.1)	2 (2.8)	1 (1.4)	0	7 (16.7)	2 (4.8)	0	0
Injection site reaction	10 (14.1)	1 (1.4)	0	0	5 (11.9)	0	0	0
Dry skin	10 (14.1)	1 (1.4)	0	0	–	–	–	–
Fatigue	9 (12.7)	3 (4.2)	0	0	10 (23.8)	4 (9.5)	0	0
Edema peripheral	9 (12.7)	1 (1.4)	0	0	2 (4.8)	0	0	0
Hot flush	8 (11.3)	2 (2.8)	0	0	3 (7.1)	2 (4.8)	0	0
Alopecia	8 (11.3)	1 (1.4)	0	0	–	–	–	–
Aspartate aminotransferase increased	8 (11.3)	4 (5.6)	1 (1.4)	0	4 (9.5)	1 (2.4)	0	0

G, grade

^a^1 patient (1.4%) experienced grade 3 febrile neutropenia in the abemaciclib arm

Four patients (5.6%) discontinued study treatment due to an AE (neutropenia, diarrhea, drug-induced liver injury, and increased aspartate aminotransferase, *n* = 1 each). Twenty-eight (39.4%) patients in the abemaciclib versus 1 (2.4%) patient in the placebo arm had at least one dose reduction in abemaciclib/placebo due to an AE. In the abemaciclib arm, the AEs leading to dose reduction included neutropenia (*n* = 14, 19.7%), diarrhea (*n* = 12, 16.9%), nausea (*n* = 2, 2.8%), and lymphopenia *n* = 1, 1.4%).

No deaths due to AEs, during or within 30 days after treatment, occurred in the premenopausal population. At the primary PFS lock, one pre-menopausal subject was reported as death (within 30 days of treatment discontinuation) due to grade 5 embolism event reported as cerebral infarction. Later, at the OS interim lock, the investigator re-evaluated the primary death reason to be study disease.

## Discussion

MONARCH 2 study reported a large absolute OS benefit in a phase III clinical trial for HR+, HER2- ABC, regardless of menopausal status [6, 16]. In this report, we evaluated the treatment benefit of abemaciclib plus fulvestrant in the premenopausal subgroup of the MONARCH 2 trial. Our findings indicate that premenopausal patients with ET-resistant, HR+, HER2- ABC derived benefit from the addition of abemaciclib to fulvestrant, with outcomes broadly consistent with those in the overall ITT population.

In this subset study, PFS was prolonged in premenopausal subgroup (HR 0.415) with the addition of abemaciclib to fulvestrant. Additionally, the observed numerical OS benefit in this subgroup (HR 0.689) was consistent with findings from the ITT population (HR 0.757).

Improvements in favor of the abemaciclib arm were observed for all exploratory endpoints including PFS2, TTC and CFS, also consistent with the ITT population [15], further strengthening our understanding of the treatment benefit with abemaciclib. The improvement in PFS2 demonstrated the treatment effect of abemaciclib carried over beyond the first disease progression to the subsequent line of therapy, which is of clinical relevance. Treatment with abemaciclib delayed chemotherapy and the median TTC was almost doubled in the abemaciclib plus fulvestrant arm compared to placebo plus fulvestrant arm. Post-discontinuation therapy was well-balanced considering the number of patients remaining on study treatment in the abemaciclib plus fulvestrant arm. The higher percentage of patients receiving a post-discontinuation therapy in the placebo arm is linked to the fact that a higher percentage of patients on the placebo arm discontinued study treatment. Of note, although the difference in median TTC is substantial, the absolute median in each arm for TTC should not be over-interpreted due to a lack of adjustment for patient death, as patients who died prior to receiving chemotherapy were censored.

The safety profile of the premenopausal subgroup was similar to that of the ITT population. Overall, treatment was well-tolerated, and diarrhea associated with abemaciclib was generally predictable (occurred early), manageable (with conventional doses of antidiarrheal medication and dose reduction), and reversible. No differences in the safety profile were observed in premenopausal women compared to postmenopausal women in the overall population.

The effect of CDK4 and 6 inhibitors on premenopausal patients with ABC has been previously studied in two other trials. The combination of ribociclib plus ET (nonsteroidal aromatase inhibitor, ortamoxifen), evaluated in the MONALEESA-7 trial in a premenopausal population, demonstrated an improved PFS (median, 23.8 vs 13.0 months; HR 0.55; *P* < 0.0001) [4] and OS (HR 0.71; 95% CI 0.54–0.95; 4-year OS follow-up, HR 0.76; 95% CI 0.61–0.96) [6, 19]. In contrast, the PALOMA 3 trial evaluating another CDK4 and 6 inhibitor, palbociclib, in combination with fulvestrant did not demonstrate an OS benefit among premenopausal patients (HR 1.07; 95% CI 0.61 to 1.86; *P* = 0.25) [16]. This finding was consistent with the results in the ITT population, where no OS benefit was observed.

Notably, patient population, inclusion criteria and prior treatments differed among the MONARCH 2, MONALEESA-7, and PALOMA-3 trials. The MONALEESA-7 trial only included premenopausal women with ≤ 1 line of prior chemotherapy for ABC and 40% had de novo metastases [4]. The PALOMA-3 trial included both postmenopausal and premenopausal women, and 75% overall had prior ET or chemotherapy for ABC (prior ET, 46.1%; prior chemotherapy, 34.0%) [5]. In the MONARCH 2 trial, postmenopausal and premenopausal women with no prior chemotherapy for ABC were enrolled, and 38% had 1 line of ET for ABC. These differences should be considered while interpreting and comparing results among these studies.

A potential limitation of this analysis is the limited sample size. Given that formal statistical testing was not planned within the premenopausal subgroup, the focus of this analysis was to estimate the key efficacy parameters and to describe the safety profile in this patient population. Therefore, these results within premenopausal patients need to be interpreted with caveats for the MONARCH 2 trial as well as in the context of any cross-trial comparisons. Nonetheless, we observed that the magnitude of the treatment benefit in the premenopausal subgroup is comparable to that in the ITT population, suggesting a clinically meaningful improvement. Follow-up is ongoing to further characterize the long-term benefit.

## Conclusion

Current clinical guidelines recommend that patients with HR+, HER2- disease should be treated preferentially with ET, and chemotherapy reserved for rapidly progressing, or symptomatic disease. There is an unmet need for defined treatment guidelines in premenopausal breast cancer patients. Consistent with the ITT population, the benefit of abemaciclib plus fulvestrant was maintained across premenopausal women with HR+, HER2- ABC in the MONARCH 2 trial. The data support extending the use of abemaciclib plus endocrine therapy for the treatment of premenopausal women with endocrine-resistant disease.

## Supplementary Information

**Additional file 1: Supplemental Table 1**. Serious adverse events in the premenopausal population of the MONARCH 2 trial.

## Data Availability

Eli Lilly and Company provides access to all individual participant data collected during the trial, after anonymization, except for pharmacokinetic or genetic data. Data are available to request 6 months after the indication studied has been approved in the USA and EU and after primary publication acceptance, whichever is later. No expiration date of data requests is currently set once data are made available. Access is provided after a proposal has been approved by an independent review committee identified for this purpose and after receipt of a signed data sharing agreement. Data and documents, including the study protocol, statistical analysis plan, clinical study report, and blank or annotated case report forms, will be provided in a secure data sharing environment. For details on submitting a request, see the instructions provided at http://www.vivli.org.

## Abbreviations

ABC: Advanced breast cancer
CDK4 and 6: Cyclin-dependent kinase 4 and 6
CFS: Chemotherapy-free survival
CI: Confidence interval
ET: Endocrine therapy
GnRH: Gonadotropin releasing-hormone
HER2-: Human epidermal growth factor receptor 2-negative
HR: Hazard ratio
HR+: Hormone receptor-positive, ITT, intent-to-treat
ITT: Intent-to-treat
ORR: Objective response rate
OS: Overall survival
PFS: Progression-free survival
PFS2: Second disease progression
TTC: Time to chemotherapy
