# Supplementary material for: Abemaciclib plus fulvestrant in hormone receptor-positive, human epidermal growth factor receptor 2-negative advanced breast cancer in premenopausal women: subgroup analysis from the MONARCH 2 trial
Source: Breast Cancer Res. 2021 Aug 23;23:87. doi: 10.1186/s13058-021-01463-2 (PMC8381581; doi:10.1186/s13058-021-01463-2)
Supplement: Supplementary file 1 — Additional file 1: Supplemental Table 1. Serious adverse events in the premenopausal population of the MONARCH 2 trial. [file 13058_2021_1463_MOESM1_ESM.docx]

Additional File 1.

Supplemental Table 1. Serious adverse events in the premenopausal population of the MONARCH 2 trial

| **Adverse events**  **(Subjects with ≥1 SAE in either arm), n (%)** | **Abemaciclib + fulvestrant**  **N=71** | **Placebo + fulvestrant**  **N=42** |
| --- | --- | --- |
| Gastrointestinal disorders | 2 (2.8) | 1 (2.4) |
| Diarrhea | 1 (1.4) | 0 |
| Vomiting | 1 (1.4) | 0 |
| Nausea | 0 | 1 (2.4) |
| Musculoskeletal and connective tissue disorders | 2 (2.8) | 1 (2.4) |
| Muscular weakness | 1 (1.4) | 0 |
| Musculoskeletal pain | 1 (1.4) | 0 |
| Bone pain | 0 | 1 (2.4) |
| Blood and lymphatic system disorders | 1 (1.4) | 0 |
| Anemia | 1 (1.4) | 0 |
| General disorders and administration site conditions | 1 (1.4) | 0 |
| Oedema peripheral | 1 (1.4) | 0 |
| Hepatobiliary disorders | 1 (1.4) | 0 |
| Drug-induced liver injury | 1 (1.4) | 0 |
| Immune system disorders | 1 (1.4) | 0 |
| Hypersensitivity | 1 (1.4) | 0 |
| Neoplasms benign, malignant and unspecified (including cysts and polyps) | 1 (1.4) | 0 |
| Benign bone neoplasm | 1 (1.4) | 0 |
| Nervous system disorders | 1 (1.4) | 0 |
| Headache | 1 (1.4) | 0 |
| Reproductive system and breast disorders | 1 (1.4) | 0 |
| Pelvic pain | 1 (1.4) | 0 |
| Vascular disorders | 1 (1.4) | 0 |
| Embolism | 1 (1.4) | 0 |
| Infections and infestations | 0 | 1 (2.4) |
| Skin infection | 0 | 1 (2.4) |

Abbreviations: N, number of subjects in safety population; n, number of subjects with a serious adverse event; SAE, serious adverse event.
